# Effects of Grazing Season on Physico-Chemical Characteristics and Fatty Acids of Nutritional Interest of Caciocavallo Palermitano Cheese

**DOI:** 10.3390/ani12050544

**Published:** 2022-02-22

**Authors:** Antonino Di Grigoli, Marialetizia Ponte, Adriana Bonanno, Giuseppe Maniaci, Marco Alabiso

**Affiliations:** Dipartimento Scienze Agrarie, Alimentari e Forestali (SAAF), Università degli Studi di Palermo, Viale delle Scienze, 90128 Palermo, Italy; antonino.digrigoli@unipa.it (A.D.G.); marialetizia.ponte@unipa.it (M.P.); adriana.bonanno@unipa.it (A.B.); marco.alabiso@unipa.it (M.A.)

**Keywords:** grazing, autochthonous cow breeds, artisanal cheese, fatty acids, nutritional indices, human health

## Abstract

**Simple Summary:**

Cheeses obtained from grazing animals are considered by attentive consumers to be qualitatively better for various reasons linked to ethical, environmental and health aspects. It is known that these cheeses have a chemical composition, especially in terms of fatty acid profile, which makes them more beneficial for human health, preventing the risk of cardiovascular diseases and cancer. On the other hand, the quantitative and qualitative productivity of the pastures is inconstant during the different seasons of the year; therefore, in extensive livestock farms there is the need to integrate the diet of lactating cows with feed integrations in some periods. The purpose of this investigation was to evaluate the quality of an artisanal cheese produced in extensive farms (11) in the different seasons of the year (summer, autumn-winter, and spring), with particular reference to those fatty acids that have relevance in determining positive effects on human health.

**Abstract:**

The aim of this work was to evaluate, in the different production seasons of the year, the physico-chemical quality of an artisanal cheese traditionally obtained from autochthonous grazing cows, with particular reference to fatty acids (FA) of nutritional interest that play an important role in the risk or prevention of some human pathologies. For this purpose, cheeses were sampled in 11 farms, repeating the samplings in 3 different periods of the year (summer, autumn–winter, and spring) when the productive conditions of the pastures varied. The cheeses produced in the spring period, when cows ingest a greater amount of grazed forage, resulted in a more adequate composition of the main FA, which are recognized as having a health effect, such as α-linolenic, trans-vaccenic, rumenic, docosapentaenoic (DPA) and docosahexaenoic (DHA) acids. Branched-chain FA were found in greater quantities in spring cheeses, as well as in summer ones. The FA composition of cheeses produced in the different seasons was reflected in some nutritional indexes that also resulted as more suitable in cheeses obtained in the spring period. The positive effects induced on the FA profile of cheeses are presumably linked to the diet of autochthonous cows, which is mainly based on forage from natural pastures. Therefore, the results obtained confirm the benefits of grazing, which is able to guarantee the production of healthier cheeses for consumers.

## 1. Introduction

Food consumption has changed considerably in recent years. Food, in addition to being the essential source of energy for living, has become a lifestyle and also a means of interaction and distinction between people. The modern consumer is more informed, critical, exigent and, with increasing health trends, stimulated by the wrong consumption models of developed countries that have generated serious health problems (obesity, cardiovascular diseases, cancer). Furthermore, due to frequent food scandals, the modern consumer has less confidence in the large industrial agri-food companies that are often the cause.

Moreover, there is an increasing interest in sustainable food, coming from the short supply chain, the so-called “0 km” products, in order to reduce the environmental impact by mitigating the CO_2_ emissions resulting from long transport [1] and also satisfy a nostalgic exigency and the need for authenticity [2].

There is also a growing interest of consumers in animal products obtained in pasture-based systems where the main purpose is not the maximization of production, as occurs in intensive-housed systems, but mainly obtaining quality products that are safe and genuine, with the maximum respect for the environment and animal welfare [3].

Many indigenous breeds, characterized by the ability to exploit natural pastures, are reared in the Mediterranean areas where they contribute to maintaining livestock activity in marginal productive contexts; therefore, these breeds play an important role in using and enhancing resources that do not directly compete with human nutrition and also in economically sustaining the local rural communities and safeguarding the territory from environmental damage [4,5,6].

The valorization of autochthonous breeds must necessarily provide the improvement of profitability of their rearing, which, in the Mediterranean countries, is often based on the enhancement of dairy products. For this reason, the feeding regime of animals is increasingly taken into consideration, since it must support the nutritional needs of the animals to maintain adequate productivity but also respect the traditional forms of the extensive farming system that in almost all periods of the year is based on grazing spontaneous vegetable resources [7].

In the Mediterranean areas, however, it is known that pasture allowance is characterized by high quantitative and qualitative fluctuations during the different seasons of the year. The production of forage biomass is highest in spring when temperature and rain conditions are optimal, stops in summer when temperatures increase, then starts again for a short period in autumn, before stopping again in winter with lowering of temperatures [8]. Consequently, livestock farmers who operate in pasture-based systems empirically modulate the feed integration administered to animals according to their physiological stage, production level and pasture intake.

As has been widely demonstrated, the feeding of grazing ruminants plays a fundamental role in improving the nutritional and health properties of dairy products. In fact, the fresh forage, ingested by grazing animals, transfers into the milk compounds able to carry out beneficial actions for human health, thus making dairy products functional foods [9,10]. In the case of animal products, these compounds are mainly represented by some molecules belonging to the classes of polyphenols and vitamins, as well as some fatty acids (FA), including the polyunsaturated FA (PUFA) of the C18 series (linoleic and α-linolenic acids), the omega-3 PUFA, and the rumenic acid; this latter is the most represented isomer of conjugated linoleic acid (CLA), recognized to have antidiabetic, antiatherogenic and anticancer effects. In addition, branched chain FA, other than having a favorable effect on the incidence of cardiovascular diseases, show high anticancer activity [11,12,13,14,15].

Therefore, it is evident that in extensive farming systems the grazing of animals can be decisive to enhance milk and cheese production, and this would help to dispel the negative perception that an increasing segment of consumers has towards dairy products, often unjustly accused of causing health problems due to their acidic composition, unbalanced towards saturated and trans fatty acids [16].

The objective of this investigation was to characterize the Caciocavallo Palermitano cheese produced with raw milk of autochthonous Cinisara cows, using artisanal methods, in relation to the different season of the year and, consequently, to the different feeding regimes applied in extensive farming systems. The cheeses have been evaluated by determining the main physico-chemical characteristics and the FA profile, with particular reference to FA of nutritional interest that play an important role in the risk or prevention of cardiovascular disease and cancer.

## 2. Materials and Methods

Caciocavallo Palermitano is usually produced by traditional small farms located in marginal areas of the coastal and inland northwestern part of the province of Palermo (Sicily, Italy) [17]. Cheeses were collected from 11 farms considered representative of the production area of Caciocavallo Palermitano cheese. All the farms reared Cinisara cows, a Sicilian autochthonous breed described in previous papers [5,18]. The altimetric distribution of the farms was very variable, ranging between 20 and 1000 m above sea level. At the time of cheeses collection, general information about each farm was recorded, focusing mainly on the feeding of lactating cows (type and management of pastures, quantity and type of feed integration received). Farms resulted almost homogeneous with regard to the cows’ feeding regime. Indeed, all the farms were characterized by the availability of natural pastures with a high slope and the presence of outcropping rocks used by the cows with rotational grazing during the different periods. These natural pastures were characterized by a high diversity of floristic composition. Botanical characterization studies carried out in the areas under investigation (unpublished data) revealed the presence of more than 350 taxa and 50 families; the most represented botanical families were Fabaceae (21%), Asteraceae (17%), Poaceae (13%), Apiaceae (6%), Brassicaceae (5%) and Malvaceae (3%). Arable land plots were available in some farms and used to obtain forage stocks in the form of hay. The grazing stocking rate (cows/ha) was always less than 2, which is the threshold indicated in the European law that regulates organic livestock. In all the farms, the lactating cows had access to pasture for 24 h daily, except in the two moments of the daily milking when they received the feed supplements. Only in winter, in correspondence of some days characterized by adverse thermo-pluviometric events, the cows were not sent to pasture. In the area under investigation, minimum and maximum recorded temperatures were respectively of 6–12 °C in winter, 10–23 °C in autumn, 11–25 °C in spring and 19–31 °C in summer; the seasonally accumulated rainfalls were respectively 280, 205, 115, e 25 mm/m^2^ in winter, autumn, spring and summer.

The farms were characterized by an average herd size of 23 lactating cows, and all of them used the pasture, supplemented with hay and concentrate, depending on the season of year, the available grazing area and the relative available biomass. The used supplements consisted of commercial compound feed and mixed hay. All farms processed the milk in their own cheese factory employing the traditional procedure to obtain Caciocavallo Palermitano cheese, using wooden utensils [19]. The management of animals was in accordance with Animal Welfare and Good Clinical Practice (Directive 2010/63/EU) and had the approval of the local Bioethics Committee (protocol number: UNIPA-CLE-Prot. 84097).

The collection of cheeses was repeated in three different periods of the year, distinguishing:

- Summer (**Su**), (July–September);
- Autumn-winter (**AW**), (November–February);
- Spring (March–May).

Since in spring the biomass on pasture was more abundant and five farms interrupted the administration of feed integration, collected cheeses were further divided into:

- Cheeses obtained from the milk of animals exclusively fed with herbage of pasture (**SpG**);
- Cheeses obtained from animals fed on pasture with additions of hay and concentrate (**SpI**).

In the farms, the collected Caciocavallo Palermitano cheeses were forms of about 10 kg, corresponding to the average standard weight of the product found on the market.

The collected cheeses were ripened up to 120 days in controlled conditions at a temperature of 16 °C and a constant humidity of 85%. The cheeses were sampled at 30, 60 and 120 days of ripening. For sampling, an aliquot of about 3 kg was removed from each cheese and used for the analyses, while the remaining part was sealed with liquid paraffin for food use to continue maturation.

The number of cheese samples were analyzed and the feeding supplements provided to the cows in the different seasons are summarized in Table 1. Considering the 11 farms investigated, only in two of them, due to the low quantity of milk produced by the few lactating cows in the period, it was not possible to collect the summer and autumn–winter samples. Thus, instead of the 33 planned, in total, 29 cheeses were collected. Each cheese sample was evaluated in 3 ripening periods, therefore 87 analyzes were carried out.

For each cheese sample, dry matter (DM), protein (N × 6.38), fat, and ash content were determined in accordance with International Dairy Federation (IDF) standards (4A:1982 [20], 25:1964 [21], 5B:1986 [22], and 27:1964 [23], respectively). Moreover, NaCl using the IDF procedure (17A:1972; [24]), and soluble nitrogen (N) value on an aqueous filtrate, using the Kjeldahl method [25], were evaluated.

The color of cheese was determined by the Minolta Chroma Meter (CR-300; Minolta, Osaka, Japan) using illuminant C. Outcomes are referred to as lightness (L*), redness (a*), and yellowness (b*), in accordance with the International Commission on Illumination (CIE) L*a*b* system. Hardness of cheese was expressed as maximum resistance to compression (compressive stress, N/mm^2^) determined using the Instron 5564 tester (Instron, Trezzano sul Naviglio, Milano, Italy).

The FA composition was determined in lyophilized cheese samples (100 mg) directly methylated with 2 mL of 0.5 M NaOCH_3_ at 50 °C for 15 min, followed by 1 mL of 5% HCl in methanol at 50 °C for 15 min [26]. 

Fatty acid methyl esters (FAMEs) were recuperated in hexane (1.5 mL). One microliter of each sample was inoculated by an autosampler into an HP 6890 gas chromatography system equipped with a flame-ionization detector (Agilent Technologies Inc., Santa Clara, CA, the United States). FAMEs of samples were split using a 100-m length, 0.25-mm i.d., 0.25-μm capillary column (CP-Sil 88; Chrompack, Middelburg, the Netherlands). The injector temperature was maintained at 255 °C and the detector temperature was maintained at 250 °C, with an H_2_ flow of 40 mL/min, air current of 400 mL/min, and a constant He flux of 45 mL/min. The preliminary oven temperature was maintained at 70 °C for 1 min, amplified at 5 °C/min to 100 °C, kept for 2 min, amplified at 10 °C/min to 175 °C, kept for 40 min, and to conclude amplified at 5 °C/min to an ultimate temperature of 225 °C and kept for 45 min. Helium, with pressure of 158.6 kPa and a flux of 0.7 mL/min (linear velocity of 14 cm/s), was utilized as the carrier gas. FAMEs hexane mix solution (Nu-Chek Prep Inc., Elysian, MN) was utilized to classify all FA. CLA isomers were identified using a commercial blend of cis- and trans 9,11- and 10,12-octadecadienoic acid methyl esters (Sigma-Aldrich, Milano, Italy). To identify some odd and branched chain fatty acids the individual standards of C15:0 iso, C15:0 anteiso, C17:0 iso, and C17:0 anteiso (Larodan Fine Chemicals AB, Malmö, Sweden) were used. To calculate total FA, C23:0 (Sigma-Aldrich) was added to every sample (4 mg/g of lyophilized cheese) as the internal standard.

The hypercholesterolemic saturated FA (HSFA, [27]), atherogenic index (AI, [28]), and thrombogenic index (TI, [29]) were calculated according to following formulas:

HSFA = C12:0 + 4 × C14:0 + C16:0;

AI = [(4×C14:0) + C16:0 + C18:0]/∑MUFA + ∑PUFAn6 + ∑PUFAn3];

TI = (C14:0 + C16:0 + C18:0)/(0.5 × MUFA + 0.5 × n6 PUFA + 3 × n3 PUFA + n3/n6).

The health-promoting index (HPI) was assessed with the formula proposed by Chen et al. [30]:

HPI = (n3 PUFA + n6 PUFA + MUFA)/(C12:0 + 4 × C14:0 + C16:0).

Considering the effects exerted by different FA on the two main causes of human mortality in industrialized countries, cardiovascular diseases and cancer, simplified indexes proposed by Renna et al. [31] were used, with the aim of verifying the level of risk and prevention for the mentioned diseases. 

The simplified indexes used were the following:

increased risk of onset of cardiovascular diseases = CVD-R = [C12: 0 + C14: 0 + C16: 0 + Σ trans-(C18: 2c9t11 + C18: 1t11)];

prevention of cardiovascular diseases = CVD-P = [C18: 1t11 + C18: 1c9 + C18: 2c9t11 + C18: 2n6 + Σ n3];

increased risk of onset of cancer diseases = C-R = [C14: 0 + C16: 0 + Σ n6];

cancer disease prevention = C-P = [C4: 0 + C15iso + C16iso + C18: 2c9t11 + C18: 1t11 + Σ n3].

To make them comparable with the values obtained by Renna et al. [31], the content of FA used were expressed in g/100 g of cheese.

The general impact of the FA composition on the two diseases was further estimated by the ratios CVD-R/CVD-P and C-R/C-P; the greater the index, the higher the impact on CVD and cancer.

Physicochemical parameters and FA profiles of cheeses were analyzed statistically using the SAS 9.2 software [32]. The fixed effects of production season (PS-(Su, AW, SpG, SpI)), ripening time (RT-(30, 60, 120 days)) and the interaction PS × RT were assessed by a MIXED model including the farm (11 levels) as a random effect. Since the effects of RT and MS × RT were not significant, the relative means of FA were not reported in the tables. Tukey’s test was used to compare the means when the effects were significant (*p* < 0.01 and *p* < 0.05).

## 3. Results and Discussion

Table 2 shows the physico-chemical parameters of cheeses sampled during different production seasons and at three ripening times.

Production season did not interfere with the chemical composition of cheese, which instead was influenced, as expected, by ripening time.

Indeed, passing from 30 to 120 days of aging, due to the loss of water, dry matter of cheese increased (*p* < 0.01) and, consequently, the NaCl content increased (*p* < 0.01), as reported in previous works for other cheeses in which moisture was indirectly connected to salt content [33,34,35,36].

The soluble nitrogen of the cheese was also influenced by ripening time (*p* < 0.01). It is well-known that the level of nitrogenous soluble compounds in cheeses derived from microbial proteolysis increases during aging and is used as an index of proteolysis and maturity of cheese [37]. The results of cheese hardness, as expected, confirmed that this parameter increases during storage (*p* < 0.01). Other authors, studying textural parameters of Caciocavallo cheese during ripening, observed that the hardness rises [6,38].

Color parameters (L*, a* and b*) of analyzed cheeses, unlike what other authors found, were not influenced by ripening time; nevertheless, an increasing trend can be observed with slight differences that did not reach a significant level, probably due to the limited duration of ripening time. Instead, color was greatly influenced by the production season. The cheeses obtained during the spring period, when the cows used more green forage at pasture, were less luminous (L*, *p* < 0.01) and with higher red (a*, *p* < 0.01) and yellow (b*, *p* < 0.01) indexes. Thus, this more intense tonality of red and yellow in the cheeses obtained in spring, when the cows were fed on pasture, as also found by other authors, is certainly the result of the higher content of carotenoids in the fresh forage ingested by grazing cows, which is transferred to milk and cheeses [39,40].

Table 3 shows the FA composition of cheeses sampled during different production seasons and analyzed at diverse ripening times. Overall, especially considering the FA that have an impact on human health, the ripening times did not significantly influence any of the FA examined, according to what was reported in the literature, while a significant influence was exerted by the production season.

Short and medium chain FA (C:4–C:14) were higher (*p* < 0.01) in spring and winter periods, when the animals were fed with green pasture and feed integration and probably received rations more suitable to their needs. This condition allowed them to achieve an adequate energy status, which led to a lower recourse to lipid reserves; on the contrary, in summer, due to the desiccation of grazing resources and in the presence of scarce feed integrations, a negative energy status often occurs in cows of autochthonous breeds reared in traditional farms. This trend, also found by other authors [41], seems to be related to the change in the diet of cows; in fact, in the summer season, the worsening pastures quality probably caused a decrease in the de novo synthesis of short and medium chain FA in the udder [42,43], and seems to have favored long-chain FA such as oleic acid, which is normally related with lipomobilization by animals [44]. Among the short-chain FA, the only one that has an impact on human health is butyric acid (C:4), the content of which does not depend on animal feeding [45]; butyric acid is recognized to have an important anticarcinogenic effect, especially in the large intestine, where it even exerts a chemotherapeutic effect in the form in which it is found in milk, i.e., a triglyceride with three molecules of butyric acid (tributyrin) [46,47].

The odd and branched chain FA content was also affected (*p* < 0.01) by the production season (Table 3). This class of FA, neglected in the past due to their low content compared to total FA, has recently been re-evaluated by the scientific community due to their intense beneficial activity on human health. As reported in the literature [15], these FA are synthesized de novo particularly by rumen cellulolytic bacteria and incorporated in their cellular lipid membrane, while they are little represented in rumen amylolytic bacteria; therefore, their variation in milk and cheese reflects the variations of rumen bacterial population [48]. The sampled cheeses showed a decrease in C13:0 iso, C13:0 anteiso, C14:0 iso, C15:0 anteiso, C16 iso, C17 anteiso, and C17:0 in winter, probably due to the change in the quality of the animals’ diet, which was richer in starch from concentrate and low in NDF fiber, and especially cellulose, from forage. These results are in line with those obtained in other experiments carried out with sheep, goats and cows where the level of branched chain FA was higher with the increase in the amount of forage in the ration [49,50,51]. On the whole, the total branched chain FA showed higher values both in cheeses produced in spring and in summer, when the animals benefited from a greater quantity of fiber from forage in the ration.

The cheese production season also had a significant influence on other FA with beneficial action on health, such as C18:3 n3 (α-linolenic acid, ALA), C18:2 c9t11 (rumenic acid, RA), C18:2 t11c15 (vaccelenic acid, VNA), and C18:1 t11 (trans vaccenic acid, TVA), which resulted in greater quantities in spring, when, in the extensive farming system used for autochthonous cow breeds, green forages of pasture are consumed in greater quantities than other periods of the year, as also found by other authors [2,10].

As widely found in the literature, the main effect of grazing on FA composition of dairy products is given by the high content of ALA in pasture forages, which is partially biohydrogenated in the rumen in TVA and partially absorbed directly in the animal’s intestine and transferred in the milk [52]. In addition, in the mammary gland, a significant amount of VA is converted into RA, through the action of the stearoyl-CoA desaturase enzyme, producing most of the RA found in milk and therefore in cheeses. Moreover, it is also possible, as hypothesized by Nudda et al. [53], that the ingestion of green forages causes the development of some bacterial species in the rumen responsible for the production of RA or responsible for the interruption of further transformation of TVA into stearic acid (C18:0), that is the final product of the ruminal biohydrogenation of ALA.

Furthermore, this investigation allowed to observe, in agreement with other authors [41,53,54], that the ALA content in cheeses decreased (*p* < 0.01) passing from spring to summer, in line with the decreasing availability and the advancement of the maturity of the pasture forages. 

Moreover, the SpG cheeses obtained in spring from animals fed exclusively on pasture showed significantly higher values (*p* < 0.05) of TVA and RA, even compared to the spring SpI cheeses obtained from animals that received feed integration in addition to grazing. This was probably due to the so-called “substitution effect” occurring in cows that receive dietary supplementation, which involves a lower intake of green forage from pasture, considered the main source of ALA and, consequently, of the related FA with the health effect produced by ruminal biohydrogenation (TVA, VNA and RA) [54,55,56].

The cheeses produced in the spring period (Table 3), when the cows consumed green forage, were also distinguished by the higher content of docosapentaenoic (DPA) and docosahexaenoic (DHA) acids, important long-chain omega-3 FA, which play an essential role in some human biological processes. The beneficial effects of human intake of DHA, which can also be produced directly by the human organism starting from ALA, as well as of DPA, have been widely demonstrated in the literature [57,58].

Similar results, with higher levels of these important essential FA on cheeses obtained when the animals were fed mainly on pasture, were found in other studies focusing on dairy products from cows [6], sheep [59] and goats [60].

Table 4 shows the main classes of FA that have nutritional and health significance for humans. The production season of cheeses had a notable impact on some categories of FA. The cheeses produced in spring, SpG and SpI, showed a more favorable composition for human health because they contained a higher content of PUFA (*p* < 0.01), total CLA (*p* < 0.01), and total omega-3 PUFA (*p* < 0.01), in accordance with what is reported in the literature in the case of animals fed with a prevalent amount of grazed forage in the ration [10,52,61]. Grazing also had favorable effects on the PUFA/SFA (*p* < 0.01) and omega-6/omega-3 ratios (*p* < 0.05); in particular, the latter always resulted below the threshold of 5 indicated by the FAO/WHO [62] and in line with those obtained by Altamonte et al. [63] on cheeses produced in spring.

Starting from the FA composition of cheeses, some health indexes were also calculated (Table 4). Significant differences (*p* < 0.01) were found for TI, which was better in cheeses SpG and SpI produced in spring, with grazing animals. The TI indicates the tendency of some specific FA to form clots in the blood vessels, so the consumption of cheeses with a lower TI decreases the incidence of cardiovascular disease; in all analyzed cheeses, the TI index resulted lower than those obtained in other experiments carried out on Friesian cows [64].

Considering the effects of FA on the main diseases causing human mortality in developed countries, cardiovascular diseases (CVD) and cancer (C), other simplified indexes were also calculated as proposed by Renna et al. [31] (Table 4).

Based on the FA composition of the cheeses analyzed, also in this case the cheeses produced in spring from grazing animals showed more adequate values for the cancer prevention (C-P) index and, therefore, on the cancer risk/cancer prevention (CR/CP) ratio. The CP and CR/CP indexes found in the cheeses showed better values than those of cheeses analyzed in different farming conditions by Renna et al. [31], whereas displayed the same trend observed for cheeses obtained with milk from grazing animals.

## 4. Conclusions

The most interesting results of this investigation, aimed to evaluate cheeses produced in extensive farms of autochthonous cows fed with pasture-based diets, concerned the effect of the production season on cheese FA composition and, consequently, on the nutritional indexes calculated to establish the healthiness of the cheese fat.

The cheeses produced in the spring period, when the cows consumed diets with a greater amount of grazed fresh forage, resulted in a more adequate composition of the main FA, which are recognized to have a health effect, such as α-linolenic, trans vaccenic, rumenic, DPA and DHA. Branched-chain FA were found in greater quantity in spring cheeses, as well as in summer, when the intake of fiber from forage by grazing cows increases. The differences in FA composition of cheeses due to the different production season influenced some nutritional indexes, such as thrombogenic index, the cancer prevention index and the cancer risk/cancer prevention ratio, which in this case produces more suitable results in the cheeses obtained in the spring period.

The positive effects induced by the spring season on the FA profile of cheeses are presumably linked to the diet of autochthonous cows, which is mainly based on fresh forage from natural pastures. Therefore, the results obtained confirm the benefits of grazing, which, in addition to being a valid tool for maintaining adequate sustainability of livestock, using resources not directly consumable by humans and guaranteeing important ecosystem services, is able to ensure the production of healthier foods to be made available for consumers who are increasingly attentive to these aspects.

The results of this research represent a contribution to enhancing the traditional typical cheeses so that they can acquire the right remuneration on the market, as well as to incentivize the farmers to maintain the practice of grazing animals, even in part-time form.

## Figures and Tables

**Table 1:** Cheese samples analyzed and feeding supplements offered to the cows in the different seasons.

	Measure Unit	Ripening (Days)	Summer (Su)	Autumn-Winter (AW)	Spring ^1^ (SpG)	Spring ^2^ (SpI)
Samples	*n.*	30	9	9	5	6
		60	9	9	5	6
		120	9	9	5	6
Concentrate	kg/day per head		3.2	3.6	0	3.4
Hay	kg/day per head		1.9	3.2	0	1.9

^1^ SpG = cheeses obtained from milk of animals that ingested exclusively herbage of pasture. ^2^ SpI = cheeses obtained from animals that used pasture with additions of hay and concentrate.

**Table 2:** Effect of production season and ripening time on chemical composition, hardness, and colorimetric parameters of cheeses.

	Ripening Time		Production Season (PS)					Significance (*p* Value)		
	(RT)		Summer (Su)	Autumn Winter (AW)	Spring ^1^ (SpG)	Spring ^2^ (SpI)	pSE ^3^	PS	RT	PS × RT
Dry matter (DM), %	30 d	61.45 B	62.20	59.70	61.10	62.80	1.390	0.0642	0.0001	0.9913
	60 d	62.31 B	63.30	61.00	61.50	63.50				
	120 d	66.94 A	67.70	64.40	65.10	66.50				
	Total		64.20	61.71	62.55	64.28				
Protein, % DM	30 d	46.70	46.50	47.30	46.90	46.00	0.952	0.2524	0.3025	0.9865
	60 d	47.46	48.30	48.20	47.00	46.30				
	120 d	46.38	46.40	46.90	46.60	45.70				
	Total		47.04	47.49	46.83	46.02				
Fat, % DM	30 d	41.22	41.00	39.90	41.70	42.20	1.256	0.5514	0.4748	0.5526
	60 d	40.22	39.70	41.50	39.70	40.10				
	120 d	40.23	38.70	41.50	40.00	40.70				
	Total		39.79	40.95	40.47	41.02				
Ash, % DM	30 d	6.97	7.54	6.90	6.20	7.22	0.431	0.0695	0.3641	0.9928
	60 d	6.80	7.59	6.94	6.17	6.80				
	120 d	7.22	8.04	6.97	6.60	7.31				
	Total		7.72	6.84	6.32	7.11				
NaCl, g/100 g	30 d	2.21 B	2.27	2.15	1.65	2.57	0.387	0.0745	0.0156	0.8239
	60 d	2.30 B	2.38	2.27	1.64	2.65				
	120 d	2.89 A	3.40	2.81	2.20	2.82				
	Total		2.67	2.40	1.82	2.71				
Soluble N % DM	30 d	0.51 Bc	0.54	0.48	0.48	0.55	0.065	0.1278	0.0001	0.9902
	60 d	0.59 ABb	0.64	0.52	0.59	0.64				
	120 d	0.69 Aa	0.71	0.65	0.72	0.73				
	Total		0.63	0.55	0.60	0.64				
Hardness, N (mm^2^)^−1^	30 d	0.52 Bc	0.53	0.56	0.47	0.52	0.086	0.0849	0.0001	0.9218
	60 d	0.63 ABb	0.59	0.70	0.55	0.68				
	120 d	0.77 Aa	0.73	0.85	0.63	0.87				
	Total		0.62	0.71	0.55	0.69				
L*, lightness	30 d	83.05	85.00	85.00	82.42	79.70	1.781	0.0011	0.1684	0.7260
	60 d	80.42	83.70	84.10	77.20	76.70				
	120 d	81.48	82.10	83.50	79.90	80.50				
	Total		83.60 A	84.21 A	79.83 B	78.96 B				
a*, redness	30 d	−4.18	−4.96	−4.18	−3.89	−3.68	0.243	0.0001	0.9612	0.4627
	60 d	−4.19	−4.58	−4.56	−4.13	−3.49				
	120 d	−4.14	−4.71	−4.24	−3.73	−3.90				
	Total		−4.75 A	−4.33 A	−3.92 B	−3.69 B				
b*, yellowness	30 d	24.18	19.50	24.40	27.80	25.00	1.258	0.0001	0.5535	0.8646
	60 d	24.83	20.40	25.00	26.90	26.90				
	120 d	25.13	20.70	26.20	26.20	27.40				
	Total		20.23 B	25.20 A	26.99 A	26.43 A				

^1^ SpG = cheeses obtained from milk of animals that ingested exclusively herbage of pasture. ^2^ SpI = cheeses obtained from animals that used pasture with additions of hay and concentrate. ^3^ pSE, pooled standard error. A, B, C Values in the same row with different superscripts differ significantly (*p* < 0.01). A, B, C values in the same column with different superscripts differ significantly (*p* < 0.01) while a, b, c differ significantly (*p* < 0.05).

**Table 3:** Fatty acid composition (g/100 g FA) of cheese produced in different seasons.

	Production Season (PS)					Significance (*p* Value)		
	Summer (Su)	Autumn Winter (AW)	Spring ^1^ (SpG)	Spring ^2^ (SpI)	pSE ^3^	PS	RT	PS × RT
C 4:0	2.88 B	3.39 A	3.37 A	3.36 A	0.142	0.0001	0.6347	0.0831
C 6:0	1.80 B	2.23 A	2.20 A	2.23 A	0.094	0.0001	0.7349	0.9425
C 7:0	0.13 B	0.12 B	0.20 A	0.22 A	0.023	0.0001	0.1076	0.0924 0.0936
C 8:0	0.99 B	1.29 A	1.36 A	1.37 A	0.070	0.0001	0.9420	0.9923
C 9:0	0.11 B	0.11 B	0.16 A	0.18 A	0.019	0.0001	0.5008	0.1058
C 10:0	1.89 Bc	2.52 Bb	2.82 Aa	2.79 Aa	0.175	0.0001	0.8805	0.9901
C 10:1	0.004 C	0.004 C	0.025 B	0.035 A	0.003	0.0001	0.5641	0.1108
C 11:0	0.29 Bc	0.34 Aab	0.37 Aa	0.32 Aba	0.020	0.0001	0.7529	0.3620
C 12:0	2.15 B	2.80 A	3.04 A	2.88 A	0.167	0.0001	0.8313	0.9934
C 13:0	0.14 Bc	0.16 ABb	0.18 Aa	0.16 ABb	0.009	0.0002	0.8934	0.9902
C 13:0 *iso*	0.06 A	0.02 B	0.05 A	0.05 A	0.015	0.0008	0.7665	0.1000
C 13:0 *anteiso*	0.05 b	0.05 b	0.07 a	0.06 ab	0.009	0.0369	0.3271	0.0254
C 12:1	0.06 b	0.08 a	0.09 a	0.07 ab	0.009	0.0075	0.5528	0.8327
C 14 *iso*	0.28 a	0.23 b	0.21 b	0.22 b	0.013	0.0001	0.6056	0.9046
C 14:0	9.09 B	9.97 A	10.2 A	9.88 A	0.318	0.0001	0.7248	0.9937
∑ C 4:0–C 14:0	19.9 B	23.3 A	24.3 A	23.8 A	0.521	<0.0001	0.7328	0.9418
C 15:0 *iso*	0.47	0.43	0.44	0.44	0.027	0.2228	0.9361	0.9929
C 15:0 *anteiso*	0.84 A	0.75 B	0.80 AB	0.77 B	0.035	0.0076	0.7749	1.0000
C 14:1 *cis*	0.84 a	0.83 a	0.84 a	0.74 b	0.051	0.0237	0.7228	0.9957
C 15:0	1.56	1.39	1.51	1.46	0.061	0.0889	0.9243	1.0000
C 15:1	0.08 B	0.07 B	0.09 A	0.10 A	0.009	0.0003	0.4252	0.5425
C 16 *iso*	0.51 Aa	0.47 ABb	0.41 Bc	0.41 Bc	0.026	0.0001	0.7639	0.9717
C 16:0	27.2 A	26.2 A	24.3 B	23.9 B	0.861	0.0001	0.5327	0.6835
C 17:0 *iso*	0.57	0.56	0.59	0.57	0.049	0.9040	0.0646	0.0985
C 17:0 *anteiso*	0.60 A	0.26 B	0.56 A	0.58 A	0.050	0.0001	0.0718	0.0974
C 16:1 *trans* 9	0.23 B	0.24 B	0.33 A	0.30 A	0.033	0.0032	0.6247	0.2249
C 16:1 *cis*	1.26 A	1.32 A	1.01 B	0.94 B	0.119	0.0001	0.2078	0.1152
C 17:0	0.99 A	0.86 B	0.85 B	0.86 B	0.050	0.0001	0.8427	0.9904
∑ Branched chain FA	3.48 A	2.87 B	3.23 A	3.22 A	0.184	0.0001	0.1315	0.3785
∑ Odd chain FA	3.12 Aa	2.73 Bb	2.90 ABb	2.82 Bb	0.142	0.0005	0.7414	0.9915
C 18:0 *iso*	0.072 A	0.044 B	0.077 A	0.062 A	0.014	0.0166	0.2664	0.6090
C 17:1 *cis*	0.33 A	0.24 B	0.24 B	0.23 B	0.021	0.0001	0.5275	0.8054
C 18:0	11.2	11.3	10.5	11.6	0.455	0.0850	0.7382	0.9904
C 18:1 *trans* 6	0.14	0.09	0.18	0.17	0.049	0.1197	0.8154	0.7463
C 18:1 *trans* 9	0.39 ABa	0.42 Aa	0.29 Cc	0.32 BCb	0.040	0.0006	0.5542	0.6852
C 18:1 *trans* 11 TVA **^4^**	2.72 Bc	2.95 Bc	4.75 Aa	3.69 ABb	0.499	0.0002	0.3037	0.1927
C 18:1 *trans* 12–14	0.26 B	0.29 B	0.36 A	0.36 A	0.333	0.0005	0.1984	0.3038
C 18:1 *cis* 6	0.49 B	0.50 B	0.94 A	0.93 A	0.082	0.0001	0.9423	0.6154
C 18:1 *cis* 9 OA **^5^**	21.3 Aa	19.6 Bb	16.7 Cd	18.3 Cc	0.756	0.0001	0.7646	0.9712
C 18:1 *cis* 10	0.57 ab	0.60 a	0.51 b	0.54 b	0.043	0.0399	0.3428	0.2092
C 18:1 *cis* 11	0.24 a	0.22 a	0.15 b	0.21 b	0.031	0.0218	0.7249	0.5556
C 18:1 *cis* 12	0.35 Cd	0.43 BCc	0.51 ABab	0.55 Aa	0.047	0.0001	0.6854	0.0764
C 18:1 *cis* 13	0.30 a	0.23 b	0.27 a	0.29 a	0.033	0.0270	0.0638	0.3685
C 18:1 *cis* 14	0.14	0.14	0.20	0.20	0.045	0.1190	0.0776	0.1872
C 18:2 *trans* 9–12	0.25 B	0.24 B	0.33 A	0.34 A	0.044	0.0078	0.0552	0.7734
C 18:2 *cis* 9 *trans* 12	0.000 B	0.000 B	0.072 A	0.067 A	0.016	0.0001	0.1738	0.3352
C 18:2 *cis* 9 *trans* 13	0.30	0.33	0.26	0.28	0.050	0.5093	0.069	0.3499
C 18:2 *trans* 11 *cis* 15 VNA **^6^**	0.29 C	0.49 B	0.90 A	0.79 A	0.096	0.0001	0.8084	0.9885
C 18:2 LA **^7^**	2.05 Aa	1.78 Bb	1.48 Bc	1.68 Bbc	0.153	0.0001	0.6874	0.5875
C 20:0	0.24	0.22	0.19	0.21	0.028	0.2060	0.5453	0.2124
C 18:3 n6	0.22 A	0.15 B	0.13 B	0.14 B	0.017	0.0001	0.3369	0.4264
C 20:1 *cis* 11	0.082 A	0.007 B	0.014 B	0.024 B	0.026	0.0012	0.4456	0.7427
C 18:3 n3 ALA **^8^**	0.88 B	0.92 B	1.39 A	1.25 A	0.129	0.0001	0.2784	0.1585
C 18:2 *cis* 9 *trans* 11 RA **^9^**	1.04 Bc	1.07 Bc	1.87 Aa	1.36 Bb	0.204	0.0001	0.4852	0.1510
Other isomers CLA **^10^**	0.13 Bc	0.15 Bc	0.44 Aa	0.31 Bb	0.047	0.0001	0.5237	0.1624
C 20:2 *cis*, *cis* n6	0.005 Dd	0.054 Cc	0.145 Aa	0.101 ABb	0.021	0.0001	0.9264	0.9934
C 22:0	0.17 A	0.11 B	0.12 B	0.13 B	0.018	0.0001	0.9752	0.7661
C 20:3 n6	0.062	0.067	0.055	0.066	0.010	0.6525	0.4027	0.3828
C 20:4 n6 AA **^11^**	0.15	0.13	0.13	0.14	0.032	0.7632	0.4534	0.5191
C 20: 5 n3 EPA **^12^**	0.084	0.141	0.085	0.081	0.071	0.6223	0.9324	0.8846
C 24:0	0.062 B	0.012 C	0.106 A	0.098 A	0.014	0.0001	0.1218	0.0771
C 22:5 n3 DPA **^13^**	0.002 B	0.000 B	0.035 A	0.055 A	0.010	0.0001	0.0643	0.1086
C 22: 6 n3 DHA ^**14**^	0.076 Bc	0.114 ABb	0.156 Aa	0.125 ABab	0.023	0.0002	0.4565	0.5894

**^1^** SpG = cheeses obtained from milk of animals that ingested exclusively herbage of pasture; **^2^** SpI = cheeses obtained from animals that used pasture with additions of hay and concentrate; **^3^** pSE, pooled standard error; **^4^** TVA = trans vaccenic acid; **^5^** OA = oleic acid; **^6^** VNA = Vaccelenic acid; **^7^** LA = linoleic acid; **^8^** ALA = α-linolenic acid; **^9^** RA = rumenic acid; **^10^** CLA = conjugated linoleic acid; **^11^** AA = arachidonic acid; **^12^** EPA = eicosapentaenoic acid; **^13^** DPA = docosapentaenoic acid; **^14^** DHA = docosahexaenoic acid. A, B, C Values in the same row with different superscripts differ significantly (*p* < 0.01) while a, b, c differ significantly (*p* < 0.05).

**Table 4:** The effect of production season of cheese on fatty acid profile (g/100 g FA) and health indexes.

	Production Season (PS)					Significance (*p* Value)		
	Summer (Su)	Autumn Winter (AW)	Spring ^1^ (SpG)	Spring ^2^ (SpI)	pSE ^3^	PS	RT	PS × RT
∑ saturated	64.3	65.7	64.5	64.7	1.336	0.3514	0.5048	0.8012
∑ monounsaturated	29.9	28.3	27.7	28.1	0.927	0.0612	0.6121	0.9911
∑ polyunsaturated	5.76 B	5.86 B	7.71 A	7.12 A	0.529	0.0001	0.3615	0.1334
∑ CLA **^4^**	1.17 C	1.22 C	2.32 A	1.67 B	0.237	0.0001	0.6410	0.1351
∑ n3	1.43 Bc	1.73 Bb	2.61 Aa	2.43 Aa	0.224	0.0001	0.7626	0.6624
∑ n6	2.75	2.45	2.43	2.59	0.174	0.0625	0.6037	0.6151
∑ n6/∑ n3	2.84 a	2.04 b	1.02 c	1.31 c	0.360	0.0001	0.9958	0.9732
∑ PUFA **^5^**/∑ SFA **^6^**	0.09 B	0.09 B	0.12 A	0.11 A	0.012	0.0001	0.3342	0.1448
HSFA **^7^**	65.6	68.7	68.2	66.1	2.230	0.1015	0.6418	0.9821
HPI **^8^**	0.53	0.49	0.48	0.51	0.030	0.1359	0.5126	0.9412
AI **^9^**	1.94	2.16	2.09	2.01	0.148	0.1128	0.6623	0.9910
TI **^10^**	2.31 A	2.34 A	1.86 B	1.95 B	0.165	0.0001	0.7328	0.8324
CVD-R **^11^**	9.41	9.36	8.62	9.11	0.638	0.4465	0.8052	0.6621
CVD-P **^12^**	7.26	6.80	6.94	7.16	0.384	0.3476	0.1442	0.9110
C-R **^13^**	10.06	9.78	9.40	9.66	0.545	0.4948	0.4583	0.8992
C-P **^14^**	2.22 C	2.46 BC	3.29 A	2.98 AB	0.209	0.0001	0.1312	0.4781
CVD-R/CVD-P	1.28	1.39	1.21	1.23	0.121	0.1682	0.6414	0.7275
C-R/C-P	4.80 Aa	4.12 Ab	2.81 Bc	3.25 Bc	0.421	0.0001	0.6084	0.5447

**^1^** SpG = cheeses obtained from milk of animals that ingested exclusively herbage of pasture; **^2^** SpI = cheeses obtained from animals that used pasture with additions of hay and concentrate; **^3^** pSE, pooled standard error; Abbreviations: SEp, pooled standard error. **^4^** CLA = conjugated linoleic acid; **^5^** PUFA = polyunsaturated fatty acid; **^6^** SFA= saturated fatty acid; **^7^** HSFA, hypercholesterolemic saturated fatty acids = C12:0 + 4xC14:0 + C16:0; **^8^** HPI, health-promoting index = (n3 PUFA + n6 PUFA + MUFA)/(C12:0 + 4 × C14:0 + C16:0); **^9^** AI, aterogenic index = [(4x14:0) + 16:0 + 18:0]/∑MUFA + ∑PUFAn6 + ∑PUFAn3]; **^10^** TI, thrombogenic index = (C14:0 + C16:0 + C18:0)/(0.5 × MUFA + 0.5 × n6 PUFA + 3 × n3 PUFA + n3/n6); **^11^** CVD-R = cardiovascular diseases-Risk = [C12: 0 + C14: 0 + C16: 0 + Σ trans-(C18: 2c9t11 + C18: 1t11)]; **^12^** CVD-P = cardiovascular diseases-prevention= [C18: 1t11 + C18: 1c9 + C18: 2c9t11 + C18: 2n6 + Σn3]; **^13^** C-R = cancer risk = [C14: 0 + C16: 0 + Σ n6]; **^14^** C-P = cancer prevention = [C4: 0 + C15iso + C16iso + C18: 2c9t11 + C18: 1t11 + Σ n3].On rows: A, B, C = *p* ≤ 0.01; a, b, c = *p* ≤ 0.05.

## Data Availability

All data included in this study are available upon request by contacting the corresponding author.
